# Supplementary figures and images for: Bioaccessibility and Bioavailability of (-)-Epigallocatechin Gallate in the Bread Matrix with Glycemic Reduction
Source: Foods. 2022 Dec 21;12(1):30. doi: 10.3390/foods12010030 (PMC9818522; doi:10.3390/foods12010030)

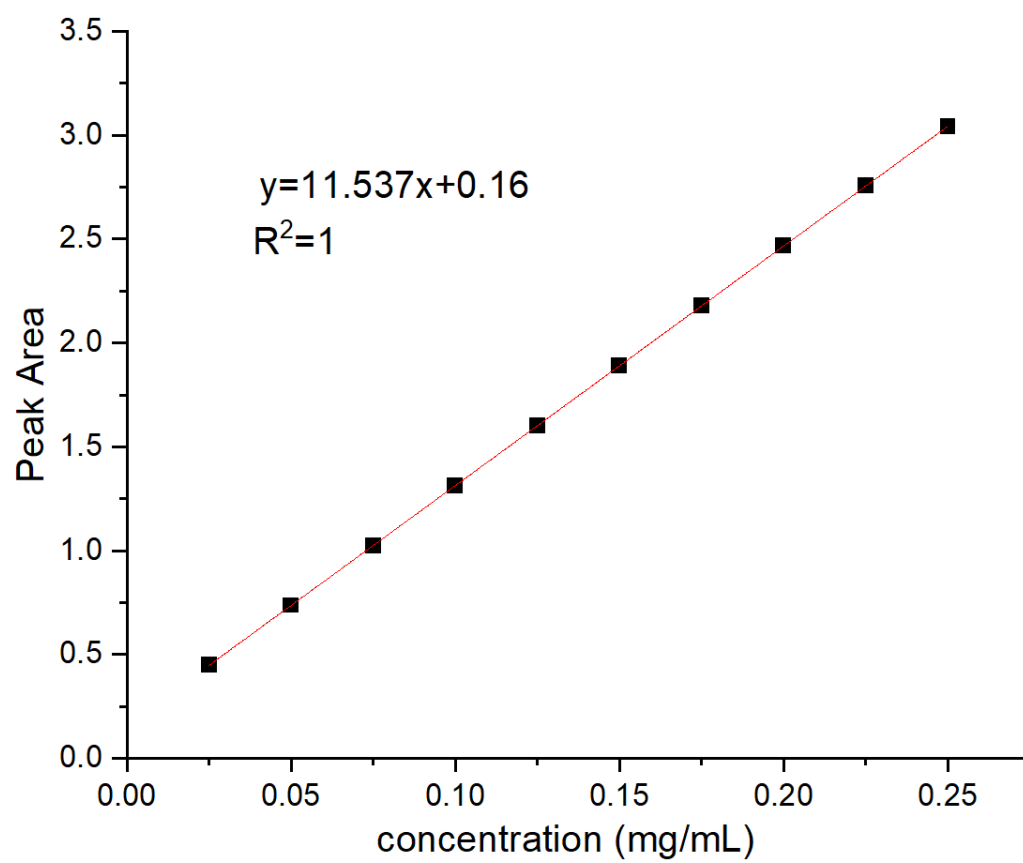

Figure S1. The calibration curve of EGCG

Supplement: Supplementary file 1 [file foods-12-00030-s001.zip › foods-2087182-supplementary.pdf]
